# Primary hyperparathyroidism associated with colorectal cancer: case report

**DOI:** 10.1093/jscr/rjad286

**Published:** 2023-05-25

**Authors:** Ahmed Y Al Ameer, Amirah Hassan Alqawba, Dalia Hamed Eid Alqarni, Reema Mesfer A Alsaluli, Adel Mohamed Aboregela, Saad A Alqarni

**Affiliations:** Department of General Surgery, College of Medicine, University of Bisha, Bisha, Saudi Arabia; College of Medicine, University of Bisha, Bisha, Saudi Arabia; College of Medicine, University of Bisha, Bisha, Saudi Arabia; College of Medicine, University of Bisha, Bisha, Saudi Arabia; Basic Medical Sciences Department, College of Medicine, University of Bisha, Bisha, Saudi Arabia; College of Medicine, University of Bisha, Bisha, Saudi Arabia

## Abstract

The association between colorectal cancer and primary hyperparathyroidism has been reported as case reports in the literature. There are few data regarding the molecular explanation of such coexistence. Here we report a case with synchronous pathologies of primary hyperparathyroidism and colorectal cancer. Furthermore, the patient has a positive family history of the same two pathologies in one of his first-degree relatives. We reviewed the literature to clarify and explain the relationship between these two diseases. We aimed to shed light on the coexistence of such conditions and to clarify if there is a relation between them or if it is just a coincidence.

## INTRODUCTION

The coexistence of primary hyperparathyroidism (PHPT) and colorectal cancer (CRC) has been reported in the literature [1]. There is no clear consensus on whether PHPT has a role in colon carcinogenesis. Here we report a case and present a literature review to shed light on the relationship between PHPT and CRC.

## CASE REPORT

In his annual checkup, a 43-year-old male intensive care physician presented to our clinic with a history of incidental findings of high calcium (2.8819 mmol/L). He underwent further investigations, showing high parathyroid hormone (PTH) levels of 21.7 pg/mL and high phosphorus (0.8548 mmol/L). Dual energy X-ray absorptiometry (DEXA) scan showed severe osteoporosis. The bone mineral density (BMD) of the lumbar spine (L1–L4) = 0.777 g/cm2 corresponds to a Z score of −3.6. The femur neck means BMD = 0.658 g/cm2, corresponding to a Z score of −2.8. The total mean dual femur BMD = 0.791 g/cm2 corresponds to a Z score of −2.0. The radius 33% BMD = 0.741 g/cm2 corresponds to Z score of 2.5. The impression is abnormal low BMD for the patient’s age and gender.

The patient underwent a parathyroid scan, which was negative for any parathyroid hot spot. During his workup, he incidentally noticed a bleeding per-rectum. After clinical evaluation by the general surgeon, the general surgeon requested a colonoscopy. The colonoscopy showed a rectal 7 mm pedunculated polyp and an ulcerated mass 2.5 cm in the rectum 6 cm from the anal verge. A biopsy of the ulcerated mass showed moderately differentiated adenocarcinoma, while the polyp was benign. The computed tomography (CT) chest, abdomen and pelvis were negative for metastasis. He underwent low anterior resection and anastomosis and uneventfully recovered. The histopathology showed moderately differentiated adenocarcinoma of atypical glands infiltrating the submucosa and minimal invasion of the muscular layer. The margins were clear, and 11 lymph nodes were negative for metastasis. His pathological staging was P T2, P N0, and the patient had no metastasis, so it was early-stage rectal cancer, and no adjuvant chemotherapy was offered. After full recovery, the patient was re-evaluated for PTH, serum calcium, phosphorus levels and PTH. Serum calcium was high, phosphorus was low and 24-h urine calcium level was 368.8 mmol/24 h. The patient realized that he had all symptoms of hypercalcemia, including bone pain, urinary frequency, the feeling of thirst, abdominal pain and constipation.

**Figure 1:**
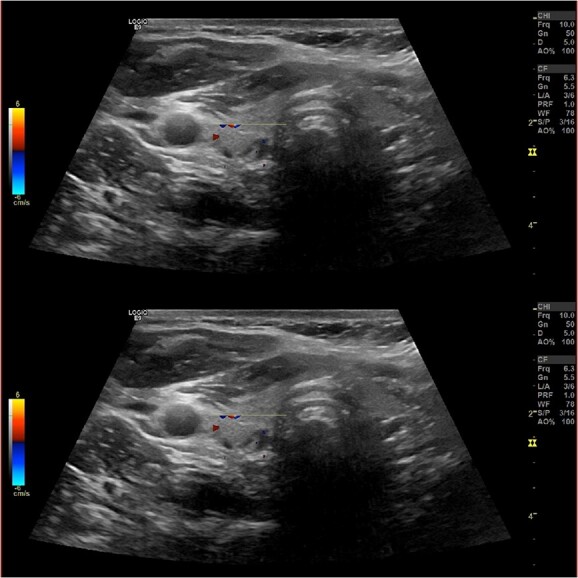
Ultrasound neck showing normal thyroid gland and no abnormal parathyroid.

**Figure 2:**
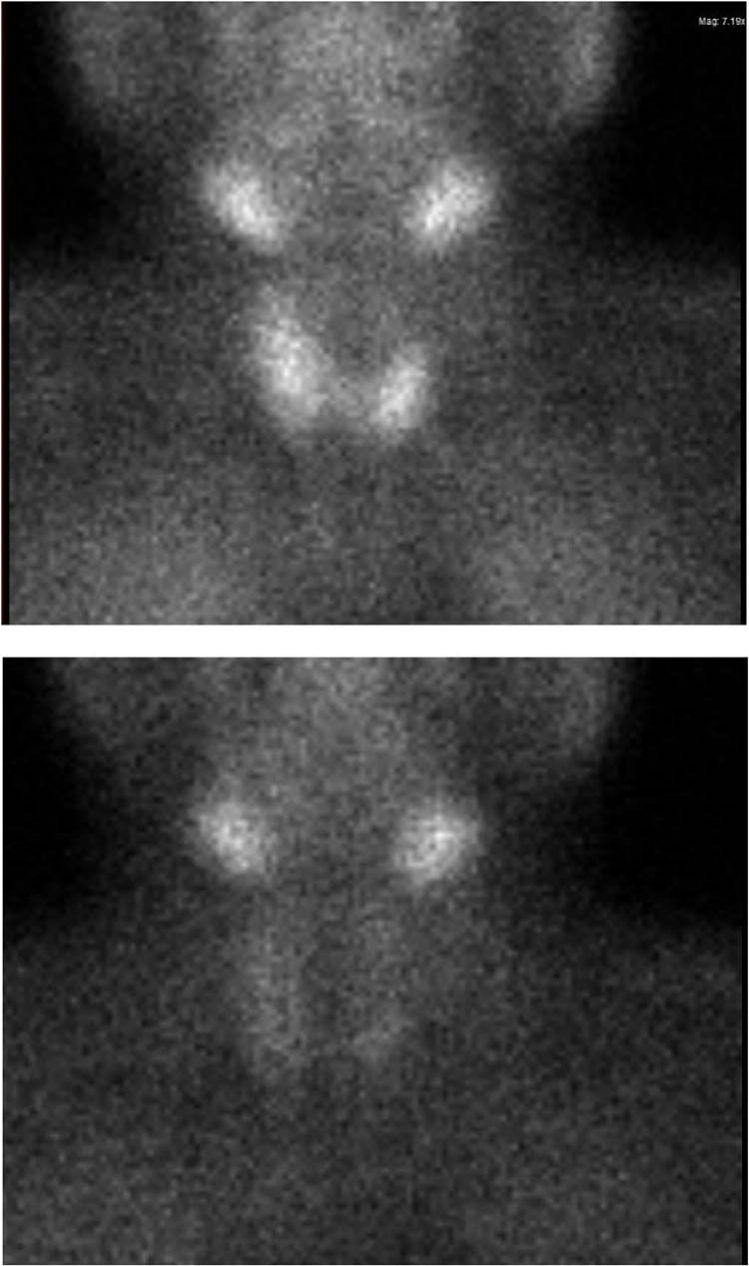
Sestambi scan showing no parathyroid uptake.

**Figure 3:**
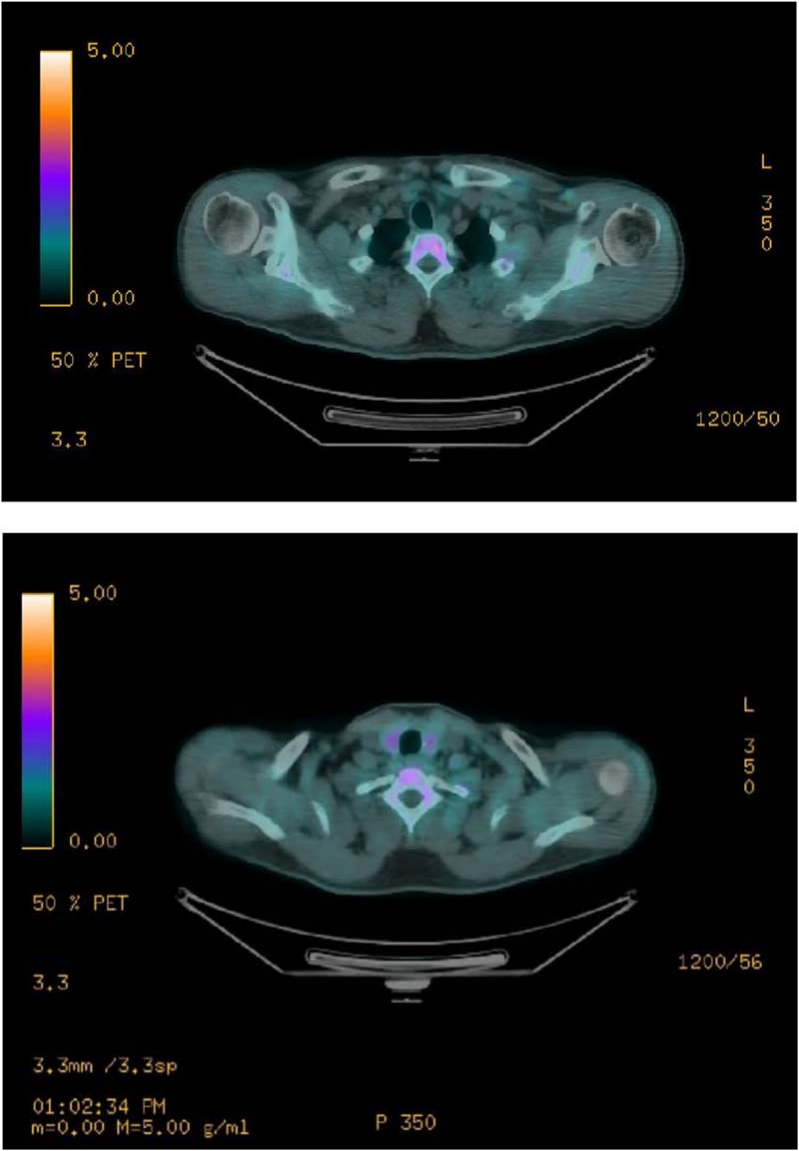
F-18 FCH PET/CT shows negative.

**Figure 4:**
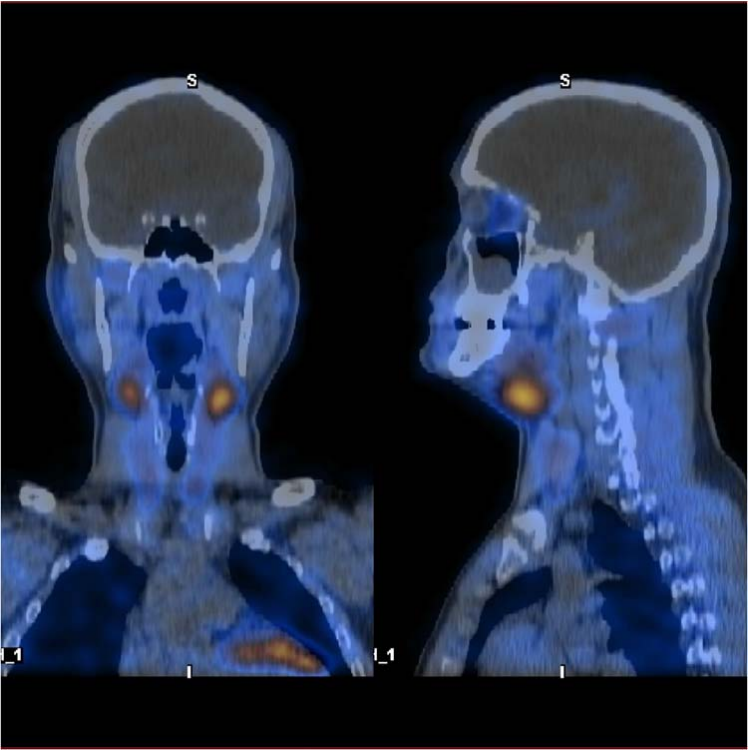
F-18 FCH PET/CT shows negative uptake.

Ultrasound of the neck (Fig. 1), CT and parathyroid sestamibi scan (Fig. 2) failed to localize parathyroid, and all studies showed normal thyroid with no other neck masses or suspicious lymph nodes. Magnetic resonance imaging neck showed no evidence of parathyroid adenoma in the expected typical gland locations or ectopic cervical or upper mediastinum. Therefore, we requested a fluorocholine (FCH) positron emission tomography (PET)/CT (Figs 3 and 4) for localization, which showed normal physiological uptake of the choline in the neck. There was no abnormal focal choline lesion in the thyroid gland or the visualized organs (unremarkable visualized part of the lungs). So, the result was a negative choline scan.

**Figure 5:**
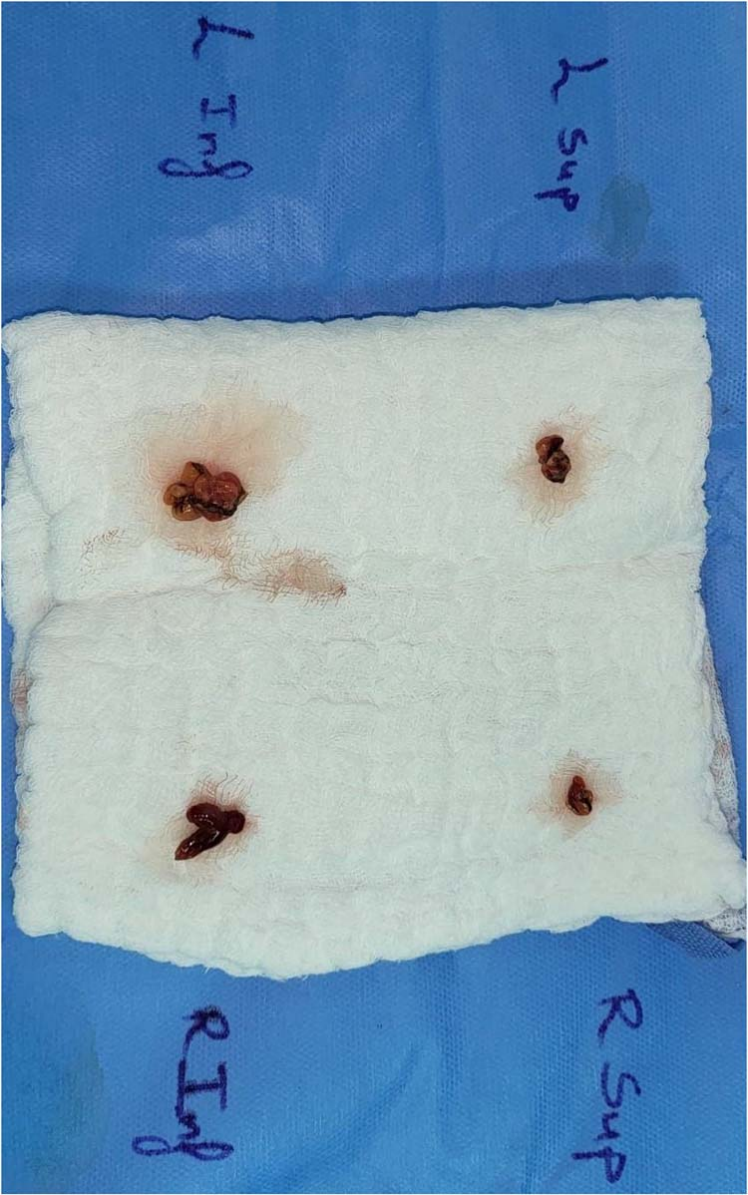
Parathyroid glands after resection.

As a multidisciplinary team (endocrinologist and endocrine surgery team), we discussed with the patient the surgical options, and we decided to take him for a neck exploration. On the neck exploration, we found four prominent parathyroid glands. A total parathyroidectomy was done, and half of the healthiest parathyroid gland was reimplanted in his right sternocleidomastoid muscle (Fig. 5). The patient postoperatively recovered uneventfully. We observed him in the intensive care unit for 24 h then we shifted him to the ward. His serial PTH and serum calcium improved gradually. After a follow-up for 1 year, all of his symptoms improved.

The histopathology showed three and a half hypercellular parathyroid glands.

## DISCUSSION

PHPT is the third most common endocrine disease. The prevalence of PHPT 1–7 /1000 adults [1, 2]. The PHPT can be classified according to etiology into sporadic (90%) and hereditary (10%). The sporadic could be due to either a single adenoma in 85% of cases or multiglandular hyperplasia in 10% of patients [3, 4]. Four per cent of sporadic PHPT is represented by double adenoma or ectopic. Parathyroid carcinoma represents 1% [5, 6]. Hereditary hyperparathyroidism (HPT) can be further subdivided into two types. Syndromic includes multiple endocrine neoplasia syndromes (MENs), MEN type 1, MEN type 2a and MEN type 4 and Hyperparathyroidism-Jaw Tumor Syndrome (HPT-JT). The second type is isolated, like Familial Hypocalciuric hypercalcemia, Neonatal Severe Primary Hyperparathyroidism or familial isolated hyperparathyroidism (FIHP) [7, 8]. From our patient workup, he likely has FIHP because he has PHPT, hypercalciuria, multiglandular hyperplasia and no features of other syndromic PHPT. The patient’s aunt has the same disease. CRC is the third most common cancer in the world [5, 9]. Sixty-five per cent of CRC cases are sporadic. Though the rest of CRC cases are inherited, only 5% have been attributed to syndromic cancer, such as hereditary nonpolyposis CRC HNPCC (2–4%) and familial adenomatous polyposis FAP (<1%). One-fourth of CRC cases have a positive family history with no apparent genetic cancer syndrome. Sporadic CRC is characterized by late onset, slowly progressing tumor. Our case showed a positive family history and early onset <50 years. Due to technical issues, we did not do a genetic test for our patient. His offspring will have >2-fold higher risk for CRCs. So, we could say it is a familial CRC, but we have no clear genetic evidence.

The coexistence of sporadic PHPT and cancers, including CRC, has been reported in the literature since the mid-1900s [4, 10]. CRC is the least frequent coexistence with PHPT among other types of cancers [11, 12]. The onset of PHPT is challenging to determine through the patient’s history because most PHPT cases (80%) are asymptomatic. The disease is diagnosed on a routine blood investigation by the presence of hypercalcemia [13, 14]. Thus, the disease consequences and complications are difficult to estimate. Based on clinical circumstances, it is difficult to answer whether sporadic PHPT contributes directly to the development of CRC from an inheritance point of view.

Furthermore, CRC is not one of the clinical features of syndromic HPT [15, 16]. CRC is common, and one can think the presentation of two common pathologies together is just a coincidence. Generally, even syndromic CRC carcinogenesis needs environmental factors that initiate and accelerate the carcinogenic process. A high-fat diet is a known risk for colonic malignancy [17, 18]. The ionized form of free fatty acid and free bile acid (the end product of a fatty diet) irritate the colonic epithelium and promote proliferation. The calcium in the crypts binds the FA and FBA and forms an insoluble soap that decreases the colonic epithelium’s exposure to FA and FBA. Hence, the low the calcium crypt level, the more crypts epithelial proliferation. At the same time, more serum PTH increases the risk of colonic malignancy through two mechanisms: decreasing calcium crypts level, acting as an oncogene, and inhibiting apoptosis [19, 20]. This basic fact explains the contribution of Hyperparathyroidism (HPTH) to colonic cancerogenesis.

## CONCLUSION

More studies must be done to explain aspects of the contribution of PHPT to CRC. More cancer screening should be provided for patients with PHPT. This relation could be an independent indication of parathyroidectomy in asymptomatic PHPT patients for the oncogenic effect of PTH and the tumor-suppressing activity of calcium.

## Data Availability

The data that support the findings of this study are available from the corresponding author [S.A], upon reasonable request.
